# Sphingosine 1-Phosphate Induces Neutrophil Chemoattractant IL-8: Repression by Steroids

**DOI:** 10.1371/journal.pone.0092466

**Published:** 2014-03-19

**Authors:** Md. Mostafizur Rahman, Hatem Alkhouri, Francesca Tang, Wenchi Che, Qi Ge, Alaina J. Ammit

**Affiliations:** 1 Faculty of Pharmacy, University of Sydney, New South Wales, Australia; 2 Woolcock Institute of Medical Research, University of Sydney, New South Wales, Australia; National Heart and Lung institute, United Kingdom

## Abstract

The bioactive sphingolipid sphingosine 1-phosphate (S1P) is found in increased amounts in the airways of asthmatics. S1P can regulate airway smooth muscle functions associated with asthmatic inflammation and remodeling, including cytokine secretion. To date however, whether S1P induces secretion of an important chemokine responsible for neutrophilia in airway inflammation – IL-8 – was unexplored. The aim of this study was to investigate whether S1P induces IL-8 gene expression and secretion to enhance neutrophil chemotaxis *in vitro*, as well as examine the molecular mechanisms responsible for repression by the corticosteroid dexamethasone. We show that S1P upregulates IL-8 secretion from ASM cells and enhance neutrophil chemotaxis *in vitro*. The corticosteroid dexamethasone significantly represses IL-8 mRNA expression and protein secretion in a concentration- and time-dependent manner. Additionally, we reveal that S1P-induced IL-8 secretion is p38 MAPK and ERK-dependent and that these key phosphoproteins act on the downstream effector mitogen- and stress-activated kinase 1 (MSK1) to control secretion of the neutrophil chemoattractant cytokine IL-8. The functional relevance of this *in vitro* data was demonstrated by neutrophil chemotaxis assays where S1P-induced effects can be significantly attenuated by pretreatment with dexamethasone, pharmacological inhibition of p38 MAPK- or ERK-mediated pathways, or by knocking down MSK-1 with siRNA. Taken together, our study reveals the molecular pathways responsible for IL-8 secretion from ASM cells in response to S1P and indicates ways in which the impact on IL-8-driven neutrophilia may be lessened.

## Introduction

Asthma can be considered a heterogeneous syndrome made up of a number of disease phenotypes characterized by their underlying pathophysiology into “asthma endotypes” [1], [2]. Defining asthma in this way will enable the design of tailored therapeutic strategies that specifically target the fundamental mechanisms responsible for the disease endotypes. One important asthma endotype is non-eosinophilic (neutrophilic) asthma. Neutrophilic asthma is driven by the chemokine CXCL8 (IL-8) [3]; thus studies into the molecular pathways that upregulate this neutrophil chemoattractant will allow us to gain greater insight into the underlying pathogenic mechanisms and suggest potential pharmacotherapeutic strategies for treating the neutrophilic asthma endotype in the future.

The causes of neutrophilic asthma are currently uncertain. Innate immunity dysregulation through TLR2 plays an important role [4], as may Th17 regulation [5] and NLRP3 inflammasome activation [6]. Activation of these cellular pathways has been reported to increase neutrophilic inflammation in the airways in an IL-8-driven manner; key characteristics of the disease endotype. Airway structural cells (such as alveolar epithelium and airway smooth muscle (ASM)) serve as important contributors to IL-8 chemokine production and in this way may orchestrate neutrophil chemoattraction in response to inflammatory mediators [7]–[9].

In this study we focus on ASM cells in order to examine whether IL-8 is produced in response to stimulation with sphingosine 1-phosphate (S1P). S1P is a bioactive sphingolipid found elevated in the airways of asthmatics [10] and can increase IL-8 secretion from human alveolar epithelial cells (A549) to regulate neutrophil–epithelial interactions *in vitro* [11]. Herein we are the first to show that S1P induces IL-8 secretion from ASM cells and demonstrate the molecular mechanisms responsible. We show that the anti-inflammatory corticosteroid dexamethasone inhibits S1P-induced IL-8 driven neutrophil chemotaxis and show that the molecular pathways responsible for corticosteroid-mediated repression converge on mitogen- and stress-activated kinase 1 (MSK1). These studies may provide further insight into the pathogenic mechanisms responsible for the neutrophilic asthma endotype.

## Materials and Methods

### ASM cell culture

Human bronchi were obtained from patients undergoing surgical resection for carcinoma or lung transplant donors in accordance with procedures approved by the Sydney South West Area Health Service and the Human Research Ethics Committee of the University of Sydney. Written informed consent from the donor was obtained for use of this sample in research. ASM cells were dissected, purified and cultured as previously described by Johnson *et al.* [12]. A minimum of three different ASM primary cell lines were used for each experiment.

### Chemicals

S1P (Biomol) was purchased through Enzo Life Sciences (Exeter, UK) and tumor necrosis factor α (TNFα) purchased from R&D Systems (Minneapolis, MN). Unless otherwise specified, all chemicals were from Sigma-Aldrich (St. Louis, MO).

### ELISAs

Cell supernatants were collected and stored at −20°C for later analysis by ELISA. IL-8 ELISAs were performed according to the manufacturer's instructions (BD Biosciences Pharmingen, San Diego, CA).

### Real-time RT-PCR

Total RNA was extracted using the RNeasy Mini Kit (Qiagen Australia, Doncaster, VIC, Australia) and reverse transcription performed by using the RevertAid First strand cDNA Synthesis Kit (Fermentas Life Sciences, Hanover, MD) as per the manufacturer's protocol. MKP-1 mRNA levels were measured using real-time RT-PCR on an ABI Prism 7500 (Applied Biosystems, Foster City, CA) with the IL-8 (Hs00174103_m1) TaqMan® Gene Expression Assay and the eukaryotic 18S rRNA endogenous control probe (Applied Biosystems) subjected to the following cycle parameters: 50°C for 2 min, 1 cycle; 95°C for 10 min, 1 cycle; 95°C for 15 s, 60°C for 1 min, 40 cycles.

### NF-κB activity assays

NF-κB activity assays were performed with the NF-κB reporter vector, pNF-κB-Luc, according to our previously published methods [7], [13].

### siRNA knock-down

ASM cells were transiently transfected using nucleofection with 1 μg MSK1-specific SMART pool siRNA as described previously [14] and cell supernatants removed for IL-8 protein measurement by ELISA and lysates utilized for MSK1 Western blotting (Cell Signaling Technology, Danvers, MA) [14].

### Neutrophil chemotaxis

Neutrophils were purified from anti-coagulated venous blood of a healthy volunteer by positive selection using magnetic-activated cell sorting (MACS) as previously described [15]. Written informed consent from the volunteer was obtained for use of this sample in research in accordance with procedures approved by the Human Research Ethics Committee of the University of Sydney. Briefly, erythrocytes were removed by dextran sedimentation followed by ficoll centrifugation to separate mononuclear cells from granulocytes. Remaining erythrocytes were lysed by osmotic shock and the pellet which contains eosinophils/neutrophils was mixed with anti-CD16 conjugated immunomagnetic beads (Miltenyi Biotec, Sunnyvale, CA) and incubated for 30 min at 4°C. The cells were then separated using MACS where the eluant was discarded. Neutrophils bound to the micro-beads were taken out of the magnetic field, flushed and prepared at a cell density of 2×10^6^/ml in DMEM+0.1% BSA to be used in chemotaxis assays. Neutrophil chemotaxis towards conditioned media from treated ASM cells was quantified microscopically using a 96-well microchemotaxis chamber (Neuroprobe, Gaithersburg, MD) as previously published [7].

### Statistical analysis

Statistical analysis was performed using Student's unpaired *t* test, one-way ANOVA then Fisher's post-hoc multiple comparison test, or two-way ANOVA then Bonferroni's post-test. *P* values <0.05 were sufficient to reject the null hypothesis for all analyses.

## Results

### The bioactive sphingolipid S1P induces secretion of IL-8 from ASM cells, but this can be repressed by the corticosteroid dexamethasone in a concentration-dependent manner

**Figure 1:**
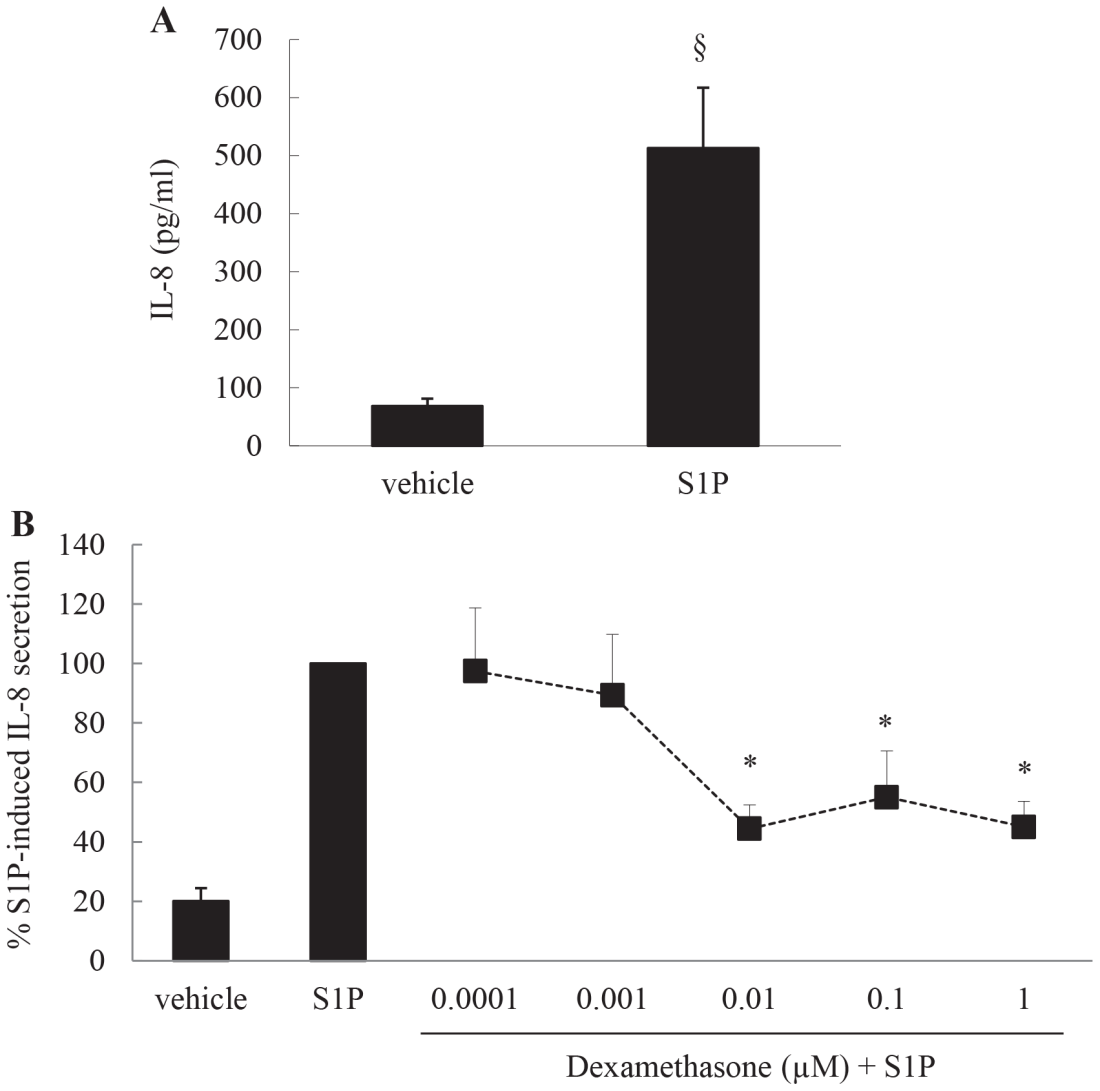
The bioactive sphingolipid S1P induces secretion of IL-8 from ASM cells, but this can be repressed by the corticosteroid dexamethasone in a concentration-dependent manner. (A) Growth-arrested ASM cells were treated with vehicle or S1P (1 μM) for 24 h and IL-8 secretion measured by ELISA. Statistical analysis was performed using the Student's unpaired *t* test, where § denotes a significant effect of S1P (*P*<0.05). (B) To demonstrate corticosteroid-mediated repression of S1P-induced IL-8 secretion we then pretreated growth-arrested ASM cells for 1 h with vehicle or dexamethasone (0.0001-1M), before stimulation for 24 h with S1P (1 μM). IL-8 protein was measured by ELISA and results expressed as % S1P-induced IL-8 secretion at 24 h. Statistical analysis was performed using one-way ANOVA then Fisher's post-hoc multiple comparison test, where * denotes significant repression by dexamethasone (*P*<0.05). Data are mean+SEM values from n = 12 primary ASM cell lines.

Our previous studies have shown that the bioactive sphingolipid S1P is increased in the airways of asthmatics [10] and as airway neutrophilia has shown to be linked to asthmatic inflammation we were interested to explore whether S1P increases secretion of the neutrophilic chemoattractant IL-8 from ASM cells. To address this, ASM cells were stimulated with 1 μM S1P for 24 h and the resultant IL-8 secretion measured by ELISA. As shown in Figure 1A, S1P significantly increases secretion of IL-8 (*P*<0.05). Importantly, S1P-increased IL-8 secretion from ASM cells can be repressed by the corticosteroid dexamethasone in a concentration-dependent manner (Figure 1B: *P*<0.05).

### Time course of S1P-induced IL-8 mRNA expression and protein secretion and its repression by dexamethasone

**Figure 2:**
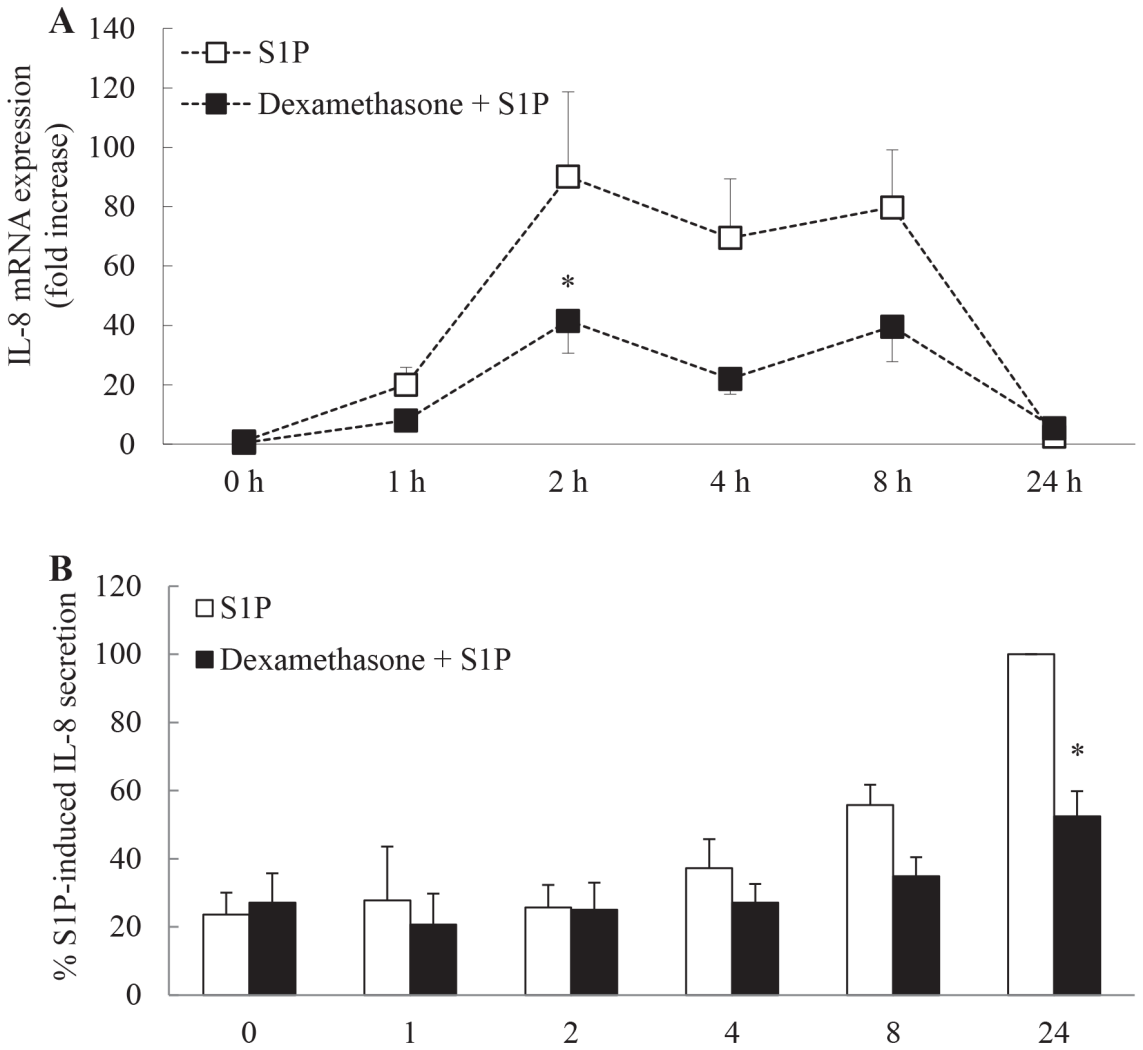
Time course of S1P-induced IL-8 mRNA expression and protein secretion and its repression by dexamethasone. Growth-arrested ASM cells were pretreated for 1 h with vehicle or 100 nM dexamethasone, followed by treatment with vehicle or S1P (1 μM) for 0, 1, 2, 4, 8, and 24 h. (A) IL-8 mRNA expression was quantified by real-time RT-PCR and results expressed as fold increase compared to vehicle-treated cells at 0 h. (B) IL-8 protein secretion was measured by ELISA and results expressed as % S1P-induced IL-8 secretion at 24 h. Statistical analysis was performed using two-way ANOVA then Bonferroni's post-test where * indicates significant repression by dexamethasone, compared to vehicle-treated cells at the same time point (*P*<0.05). Data are mean+SEM values from n = 7 primary ASM cell lines.

In order to explore the molecular mechanism of repression by dexamethasone we first performed an analysis of the temporal kinetics of S1P-induced IL-8 mRNA expression and subsequent protein secretion and its repression by dexamethasone. As shown in Figure 2A, S1P robustly increases IL-8 mRNA expression as early as 1 h after stimulation. This expression is sustained from 2-8 h, before receding to baseline levels by 24 h (*P*<0.05). This upregulation is repressed by dexamethasone pretreatment with S1P-induced IL-8 mRNA expression being significantly inhibited by dexamethasone at 2 h (*P*<0.05). The pattern of protein secretion follows the same course with significant amounts of IL-8 being secreted from ASM cells by 8 h and accumulating further at 24 h (Figure 2B: *P*<0.05). Secretion of IL-8 was substantially and significantly repressed by the corticosteroid dexamethasone at 24 h (Figure 2B: *P*<0.05).

### Dexamethasone totally inhibits S1P-induced IL-8 protein secretion, but only partially inhibits TNFα-induced IL-8 induced in the presence of S1P

**Figure 3:**
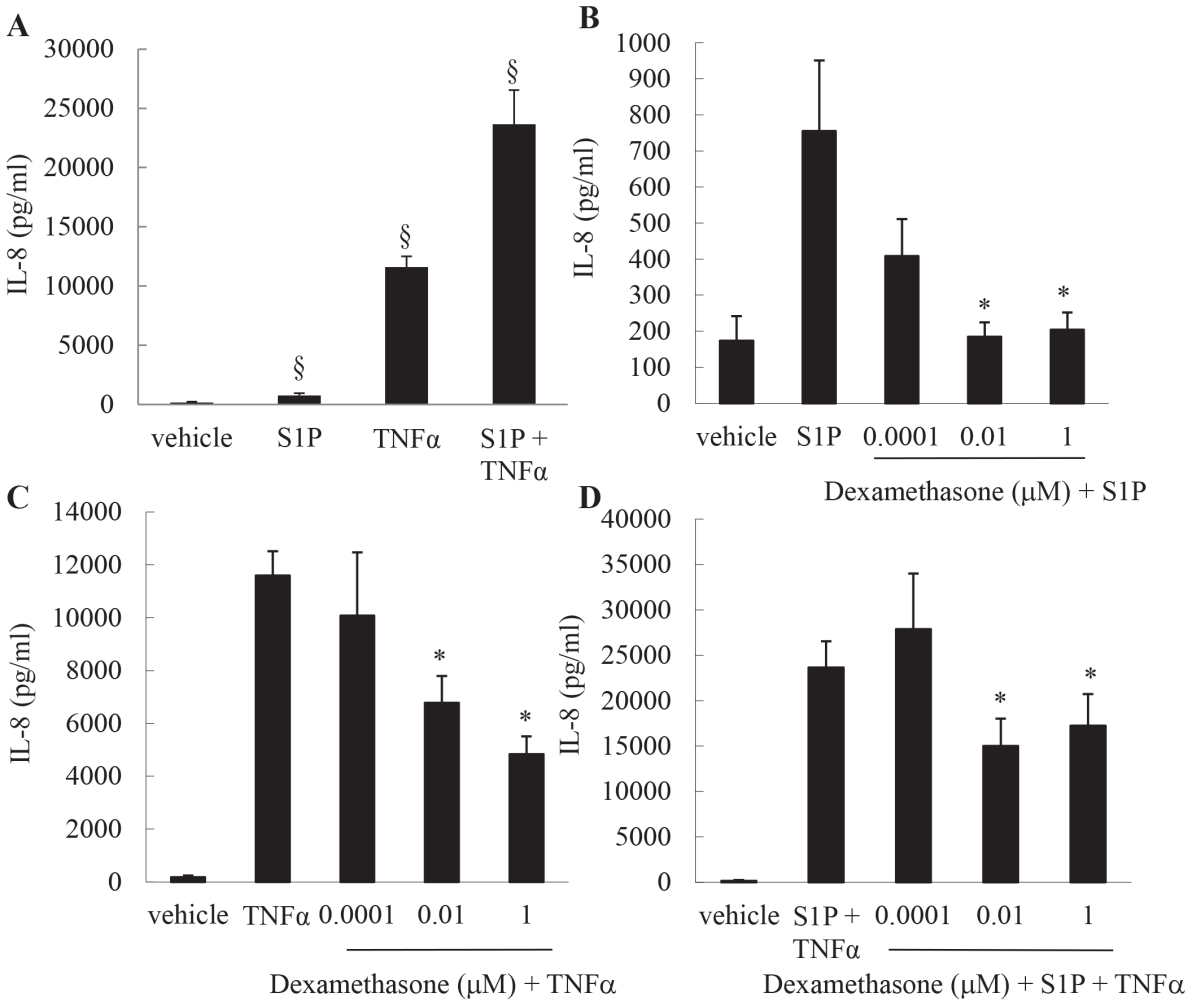
Dexamethasone totally inhibits S1P-induced IL-8 protein secretion, but only partially inhibits TNFα-induced IL-8 induced in the presence of S1P. (A) To compare IL-8 secretion, growth-arrested ASM cells were treated with vehicle, S1P (1 μM), TNFα (10 ng/ml), or S1P (1 μM) in combination with TNFα (10 ng/ml). (B–C) To examine the relative corticosteroid sensitivity, cells were pretreated for 1 h with vehicle or dexamethasone (0.0001, 0.01, 1 μM) then: (B) stimulated with S1P (1 μM); (C) stimulated with TNFα (10 ng/ml); (D) stimulated with S1P (1 μM) +TNFα (10 ng/ml). After 24 h, secreted IL-8 was measured by ELISA. Statistical analysis was performed using one-way ANOVA then Fisher's post-hoc multiple comparison test, where § denotes a significant upregulation of IL-8, and * denotes a significant effect of dexamethasone on IL-8 secretion (*P*<0.05). Data are mean+SEM values from n = 7 primary ASM cell lines.

As we had observed (in Figures 1 & 2) that dexamethasone represses S1P-induced IL-8 expression relatively effectively, S1P secretion alone cannot solely account for the more corticosteroid insensitive phenotype associated with neutrophilic asthma. As numerous inflammatory mediators can be found elevated in asthma at the same time, it is of interest to examine the relative corticosteroid sensitivity of IL-8 induced by another inflammatory mediator added in combination with S1P. TNFα is a key example [16]. Therefore we stimulated cells with S1P+TNFα in order to determine if the model is more corticosteroid resistant under these experimental conditions. We first performed a side-by-side comparison of IL-8 secretion in response to S1P or TNFα, added separately or in combination. As shown in Figure 3A, and in confirmation of our earlier studies [7], TNFα potently and significantly increases IL-8 secretion. Notably, TNFα-induced IL-8 secretion is significantly potentiated in the presence of S1P (Figure 3A: *P*<0.05). We then compared the relative corticosteroid sensitivity of IL-8 induced by each stimulant, alone or in combination. As shown in Figure 3B, IL-8 induced by S1P can be completely repressed by dexamethasone. Dexamethasone concentrations as low as 0.01 μM can repress S1P-induced IL-8 secretion to unstimulated levels. In contrast, TNFα-induced IL-8 cannot be is relatively resistant to repression by dexamethasone (Figure 3C). Even after 1 μM dexamethasone pretreatment, TNFα-induced IL-8 secretion cannot be completely repressed (Figure 3C). Accordingly, when then the two pro-inflammatory mediators are added together, S1P in addition to the potent pro-inflammatory cytokine TNFα induces IL-8 expression that is relatively corticosteroid resistant (Figure 3D).

### S1P does not activate NF-κB in ASM cells

**Figure 4:**
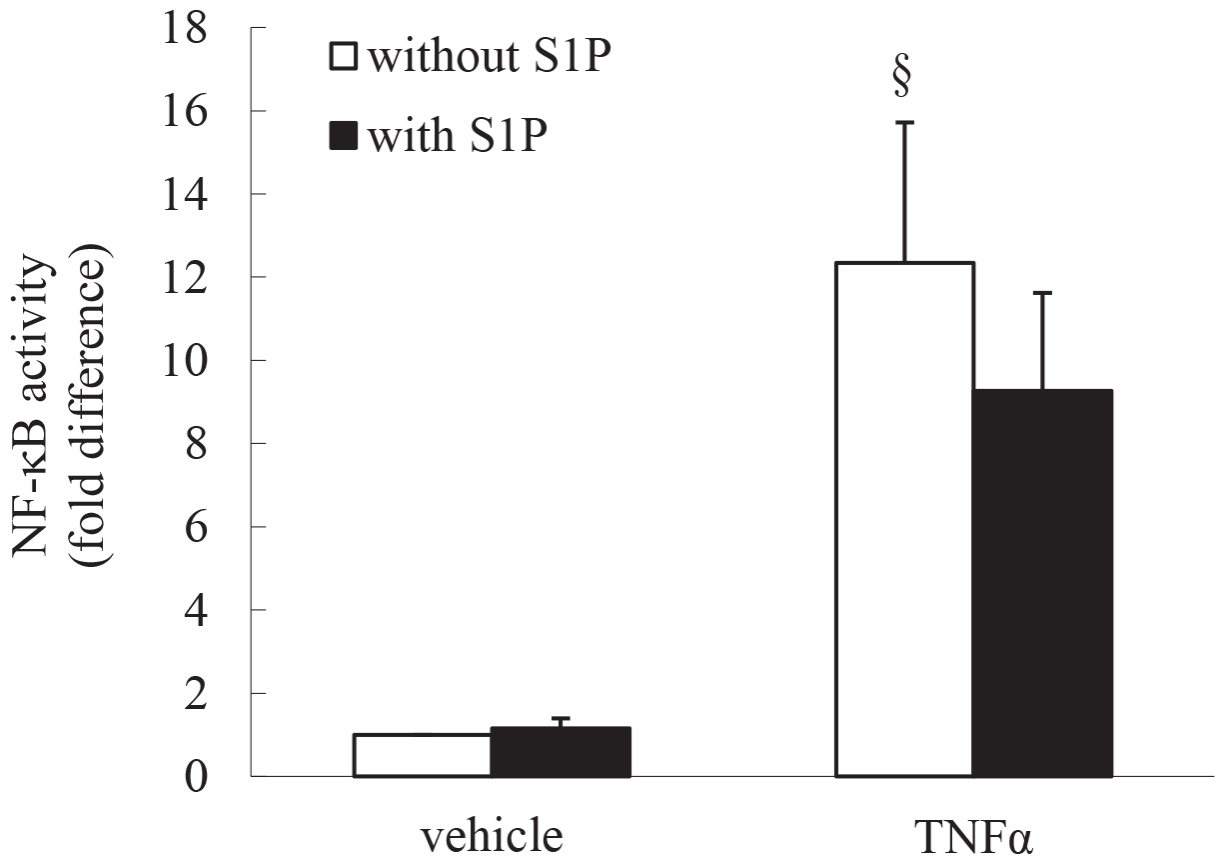
S1P does not activate NF-κB in ASM cells. ASM cells transfected with a NF-κB reporter vector, pNF-κB-Luc, were growth-arrested, then treated with vehicle or TNFα (10 ng/ml), in the absence or presence of S1P (1 μM) for 6 h. Cells were then harvested and luciferase and β-galactosidase activities assessed. Data represent normalized luciferase activity, relative to vehicle-treated cells (expressed as fold difference). Statistical analysis was performed using the Student's unpaired *t* test (where § denotes significant effect of TNFα on NF-κB activity (*P*<0.05)). Data are mean+SEM values from n = 6 primary ASM cell lines.

In order to examine whether the difference in corticosteroid sensitivity between mediators is due to the involvement of NF-κB signaling pathway in mediating IL-8 expression we utilized an NF-κB reporter vector, pNF-κB-Luc. As shown in Figure 4, TNFα activates NF-κB; in confirmation of our earlier studies [17]. Importantly, S1P alone does not increase NF-κB-luciferase activity, nor increase TNFα-induced NF-κB activity. As NF-κB activity in ASM cells is relatively insensitive to repression by corticosteroids [18]–[20], our *in vitro* results may reflect the *in vivo* situation where multiple inflammatory mediators orchestrate chemokine expression and is consistent with airway neutrophilia being corticosteroid resistant.

### S1P-induced IL-8 protein secretion is repressed by inhibitors of the p38 MAPK- and ERK-mediated pathways

**Figure 5:**
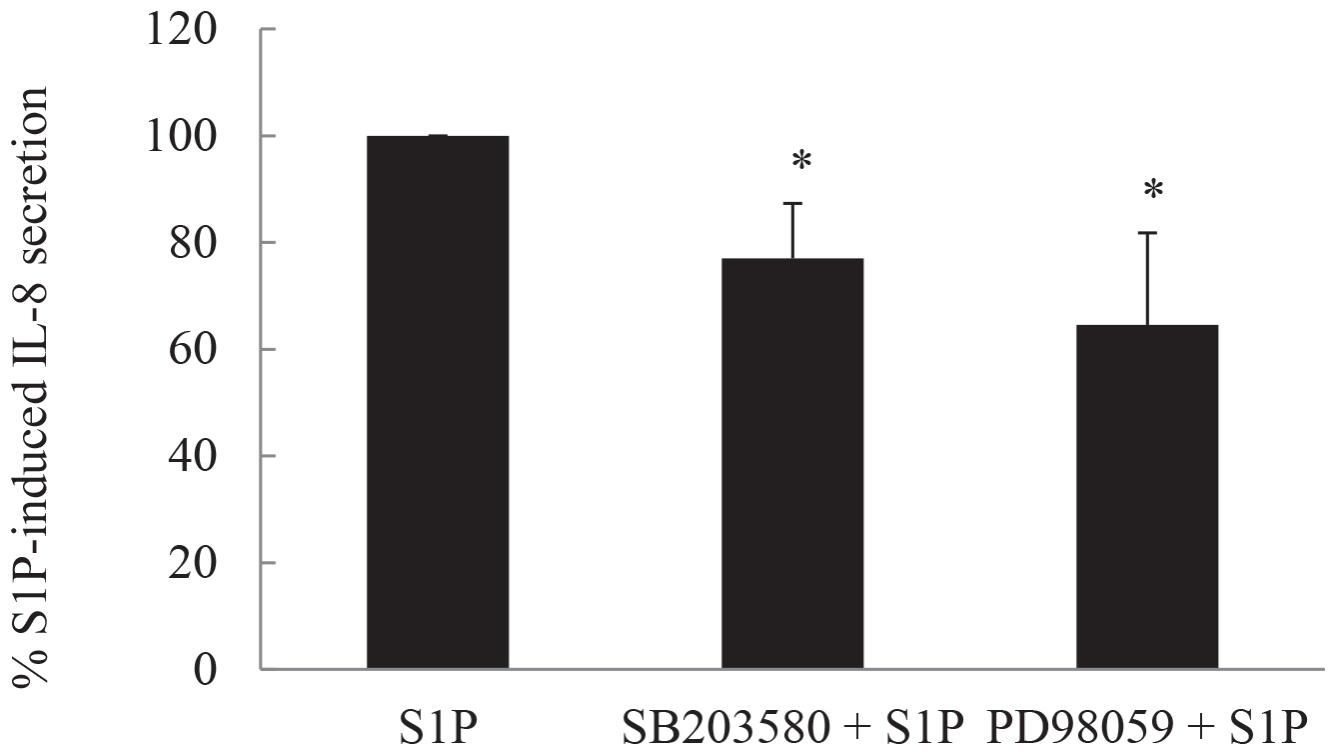
S1P-induced IL-8 protein secretion is repressed by inhibitors of the p38 MAPK- and ERK-mediated pathways. Growth-arrested ASM cells were pretreated for 30 min with vehicle, 1 μM SB203580, or 10 μM PD98059 to inhibit p38 MAPK and ERK, respectively. Cells were then stimulated with 1 μM S1P for 24 h. IL-8 protein was measured by ELISA and results expressed as % S1P-induced IL-8 secretion at 24 h. Statistical analysis was performed using the Student's unpaired *t* test, where * denotes significant inhibition (*P*<0.05). Data are mean+SEM values from n = 10 primary ASM cell lines.

We had previously shown that S1P rapidly activates all members of the MAPK superfamily [21] and had earlier implicated p38 MAPK and ERK pathways in IL-8 secretion in response to another stimulus – TNFα [13]. For these reasons we now explored the role of the p38 MAPK and ERK pathways in S1P-induced IL-8 secretion. We utilized well-established pharmacological inhibitors of p38 MAPK and ERK, i.e. 1 μM SB2035680 and 10 μM PD98059, respectively, and investigated their repressive effects on S1P-induced IL-8 protein secretion from ASM cells. We routinely utilize these inhibitors to repress p38 MAPK and ERK signaling in ASM cells *in vitro* [7], [13], [14], [20], [22], [23] as they specifically inhibit p38 MAPK and ERK phosphorylation in ASM cells at these concentration [21]. As shown in Figure 5, S1P-induced IL-8 protein secretion was significantly repressed by both SB203580 and PD98059 pretreatment (*P*<0.05).

### IL-8 induced by S1P causes human neutrophil chemotaxis *in vitro* and this can be repressed by corticosteroids or by blocking the p38 MAPK- or ERK-mediated pathways

**Figure 6:**
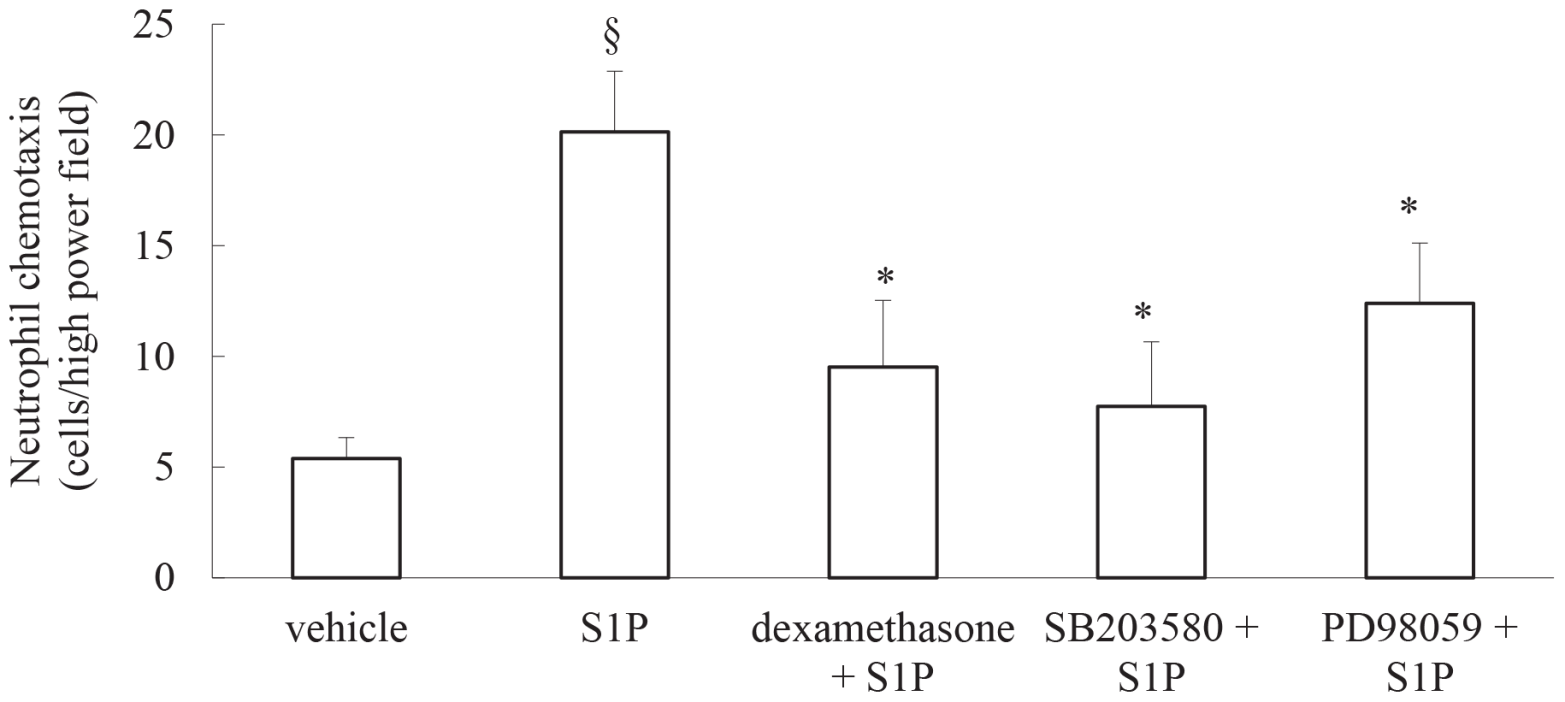
IL-8 induced by S1P causes human neutrophil chemotaxis *in vitro* and this can be repressed by corticosteroids or by blocking the p38 MAPK- or ERK-mediated pathways. Chemotaxis of human neutrophils toward conditioned media from growth-arrested ASM cells pretreated with vehicle, dexamethasone (100 nM), SB203580 (1 μM) or PD98059 (10 μM), followed by treatment with vehicle or S1P (1 μM) for 24 h, was measured using microchemotaxis chambers. Results are expressed as cells per high-power (x200) field. Statistical analysis was performed using the Student's unpaired *t* test where § denotes a significant effect of S1P on neutrophil chemotaxis, while * denotes significant repression (*P*<0.05). Data are mean+SEM values using conditioned media from n = 3 primary ASM cell lines.

Taken together, our results thus far demonstrate that S1P induces IL-8 secretion from ASM cells and that this secretion can be repressed by the corticosteroid dexamethasone and pharmacological inhibitors of the mitogen- and stress-activated protein kinases; p38 MAPK and ERK. IL-8 is a key neutrophil chemoattractant cytokine and in Figure 6 we show that human neutrophils undergo significant chemotaxis towards conditioned media from cells stimulated with S1P. Importantly, this S1P-induced chemotaxis was significantly reduced when cells were pretreated with dexamethasone, SB203580 or PD98059 prior to S1P stimulation (Figure 6: *P*<0.05). These data suggest that the bioactive sphingolipid S1P may play a role in IL-8-driven neutrophilic inflammation and implicate molecular pathways that may be targeted to reduce expression of this chemokine.

### S1P induces IL-8 secretion via a MSK1-dependent pathway and MSK1 knock-down by siRNA attenuates S1P-induced neutrophil chemotaxis

**Figure 7:**
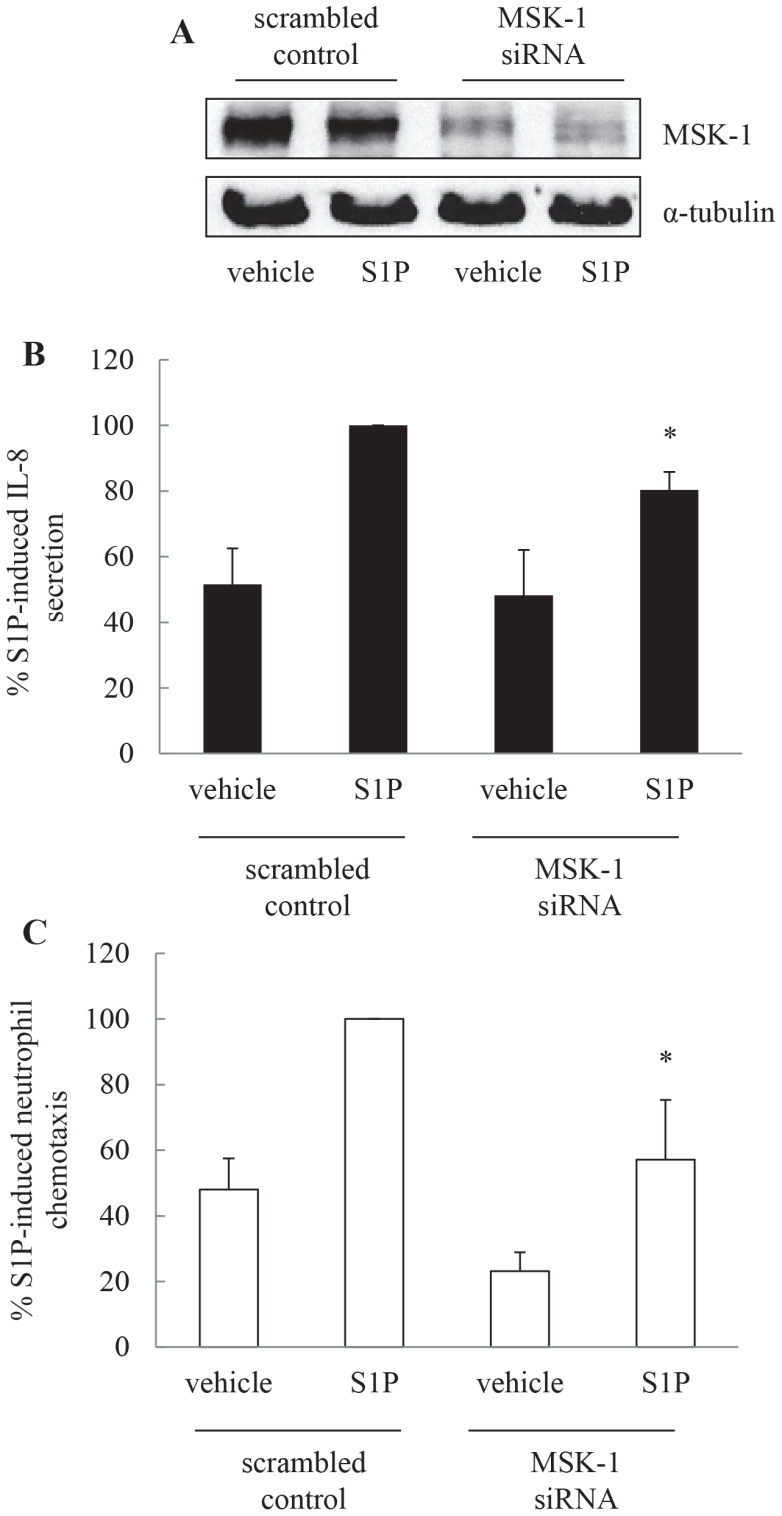
S1P induces IL-8 secretion via a MSK1 dependent pathway and MSK1 knock-down by siRNA attenuates S1P-induced neutrophil chemotaxis. ASM cells were transiently transfected using nucleofection with scrambled control or MSK1 siRNA, growth-arrested, then treated for 24 h with vehicle or S1P (1 μM). (A) To confirm that MSK1 siRNA reduces protein levels of MSK1, cells were lysed and immunoblotted for MSK1, using α-tubulin as the loading control. To measure the effect of MSK1 knock-down on IL-8 inducibility and neutrophil chemotaxis, supernatants were removed and (B) IL-8 protein measured by ELISA and (C) neutrophil chemotaxis assessed using microchemotaxis chambers. Results are expressed as: (A) representative Western blots; (B) % S1P-induced IL-8 secretion in cells transfected with scrambled control; or (C) % S1P-induced neutrophil chemotaxis in the corresponding conditioned media (data are mean+SEM values from n = 6 primary ASM cell lines). Statistical analysis was performed using the Student's unpaired *t* test where * indicates that knocking down MSK-1 significantly attenuates S1P-induced effects (*P*<0.05).

S1P-induced IL-8 is mediated via p38 MAPK and ERK-mediated pathway. One of the important downstream effectors of these mitogen- and stress-activated kinases is MSK1. We have recently demonstrated that S1P activation leads to MSK1 phosphorylation in ASM cells [14]. Thus, to address whether S1P-induced IL-8 secretion is mediated via a MSK1-dependent pathway we transiently transfected ASM cells using nucleofection with scrambled control or MSK1 siRNA and assessed the impact of reduced MSK1 on IL-8 secretion induced by S1P. Figure 7A demonstrates successful knock-down of MSK1 with siRNA. As shown in Figure 7B, the degree of IL-8 secretion was significantly less in cells transfected with MSK1 siRNA as compared to scrambled control (*P*<0.05). Accordingly, by reducing S1P-induced IL-8 secretion MSK1 knock-down also reduces S1P-induced neutrophil chemotaxis. This is demonstrated in Figure 7C, where neutrophil chemotaxis in supernatants from cells nucleofected with MSK1 siRNA was 57.2±18.2% when compared to supernatants from scrambled control cells (designated as 100%)(*P*<0.05).

## Discussion

S1P is found elevated in airways of asthmatics and in this study we explore its stimulatory effect on an important chemokine responsible for neutrophilia in airway inflammation – IL-8. We demonstrate that stimulation of ASM cells with S1P results in IL-8 gene expression and protein secretion. We determine the temporal kinetics of IL-8 upregulation and show that mRNA expression and protein secretion can be repressed by the corticosteroid dexamethasone. Additionally, we reveal that S1P-induced IL-8 secretion is p38 MAPK- and ERK-dependent and that these key phosphoproteins act on the downstream effector MSK1 to control secretion of the neutrophil chemoattractant cytokine IL-8. We demonstrate the functional relevance of our *in vitro* data by performing neutrophil chemotaxis assays. We show that S1P-induced effects can be significantly attenuated by pretreatment with dexamethasone, pharmacological inhibition of p38 MAPK- or ERK-mediated pathways, or by knocking down MSK-1 with siRNA. Taken together these studies help us appreciate the molecular pathways responsible for IL-8 secretion from ASM cells in response to S1P and indicate ways in which its impact on IL-8-driven neutrophilia may be repressed.

S1P is increasingly recognized as playing an important role in asthma and airway inflammation. We were the first to show that the levels of this bioactive sphingolipid were increased in the broncho-alveolar lavage fluid of allergic asthmatics [10] and since that time the important immunomodulatory role for S1P in asthma and airway remodeling has clearly emerged (reviewed in [24]–[26]). The source of S1P in allergic asthma is considered to be the mast cell [25], [27]. This is of relevance to the current study, where we focus on the immunostimulatory effects of S1P on airway structural cells, because we have previously shown that increased numbers of mast cells are close proximity to ASM in airways of sensitized individuals [28]; a finding confirmed in the airways of asthmatics [29]. These studies suggest that S1P released from mast cells may act upon the surrounding airway tissue to initiate, perpetuate or perhaps amplify airway responses, including cytokine secretion. In support, we have previously shown that S1P potently induces secretion of IL-6 from ASM [10], [14] and herein show that expression of IL-8 is also stimulated by S1P. Our study is in accord with a recent publication that demonstrated that S1P also enhanced IL-8 secretion from alveolar epithelial cells [11]. These studies suggest that the bioactive sphingolipid S1P can orchestrate cytokine production in airway inflammation and in the current study we extend our investigation to demonstrate that S1P can enhance neutrophil chemoattraction. This study is of clinical relevance given the contribution of airway neutrophilia to asthma pathophysiology.

Our recent papers in ASM cells have highlighted some of the key signaling pathways activated by S1P in ASM cells. S1P rapidly activates all members of the MAPK superfamily and also induces robust and sustained phosphorylation of the cAMP-dependent transcription factor CREB [14], [21]. Taken together with the knowledge that IL-8 secretion from ASM cells can be inhibited by SB203580 and PD98059 [13], but is independent of the cAMP/CREB-mediated pathway [30], we were able to narrow our focus for investigation in this study and reveal for the first time that S1P-induced IL-8 secretion is mediated by p38 MAPK- and ERK-dependent pathways. This is important as we have recently shown that the downstream effector of these mitogen- and stress-activated protein kinases – MSK1 - plays an essential role in controlling histone H3 phosphorylation, enhanced chromatin relaxation and regulation of gene expression. Thus, by stimulating p38 MAPK/ERK, S1P induces IL-8 expression in ASM cells in an MSK1-dependent pathway. This was confirmed by knocking-down MSK1 with siRNA and showing that S1P-induced IL-8 secretion was attenuated.

Our study also reveals that the corticosteroid dexamethasone can reduce S1P-induced IL-8 gene expression, and resultant protein secretion, in a time- and concentration-dependent manner. Our recent study [14] explored the signaling pathways responsible for secretion of another cytokine released by ASM cells after S1P stimulation, namely IL-6, and revealed how corticosteroids act to exert their anti-inflammatory effects. We know now that corticosteroids mediate their anti-inflammatory actions, in part, via upregulation of the anti-inflammatory protein-MKP-1 (mitogen-activated protein kinase phosphatase 1) [14], [20], [31]. Importantly, inhibition of the MSK1/histone H3 pathways is one of the key ways in which corticosteroids mediate their repressive effects in ASM cells. This was shown in our recent study [14] where corticosteroid-induced MKP-1 attenuated S1P-induced IL-6 secretion by dephosphorylating p38 MAPK and ERK-mediated activation of MSK1 and histone H3 phosphorylation. We propose that S1P-induced IL-8 is controlled in a similar MSK1-dependent manner. We illustrate the clinical relevance of targeting MSK1 by demonstrating that knocking-down MSK1 significantly represses IL-8-driven neutrophil chemotaxis induced by S1P. Thus MSK1 inhibition may represent a feasible therapeutic option for controlling S1P-mediated pro-inflammatory actions in the future.

The role of neutrophils in airway inflammation has recently come to the fore and therefore in this study we examined whether the asthma-related sphingolipid S1P increased secretion of the neutrophil chemoattractant IL-8 from ASM cells. Demonstration of S1P-induced neutrophil chemotaxis is clinically relevant given the important role played by IL-8-driven airway neutrophilia. Interestingly, the degree of reduction in neutrophil chemotaxis achieved with the MAPK pharmacological inhibitors is greater than the attenuation of IL-8 secretion. This may indicate that other MAPK-dependent chemokines may also be upregulated by S1P. Although IL-8 is the classical chemotaxin for neutrophils, other chemokines such as GROα (CXCL1) and regulated on activation normal T cell expressed and secreted (CCL5: also known as RANTES) can also contribute to airway neutrophilia [32]. These chemokines can be secreted by ASM cells [7], [17], [33], [34] and their expression is regulated by p38 MAPK/ERK- dependent pathways [7], [34], [35]. We are the first to reveal the signaling pathways activated by S1P in ASM cells [14], [21], but based on our demonstration that S1P induces IL-8 to enhance neutrophil chemotaxis in a p38 MAPK/ERK-manner we predict that other chemokines regulated by these signaling molecules may repressed by pharmacological inhibition in a similar manner. This study may have implications for revealing the pathogenic mechanisms responsible for neutrophilic asthma endotype, although to date, whether S1P levels are increased in BAL fluids from patients characterised with neutrophilic asthma is an open question.

Together, these studies indicate that inflammatory mediators such as S1P may contribute to the development of IL-8-driven neutrophilia in airway inflammation and suggests that further studies into the causal role played by S1P in the neutrophilic asthma endotype are warranted.
